# Point Cloud Resampling by Simulating Electric Charges on Metallic Surfaces

**DOI:** 10.3390/s21227768

**Published:** 2021-11-22

**Authors:** Kyoungmin Han, Kyujin Jung, Jaeho Yoon, Minsik Lee

**Affiliations:** 1 Department of Electrical and Electronic Engineering, Hanyang University, 55 Hanyangdaehak-ro, Sangnok-gu, Ansan-si 15588, Gyeonggi-do, Korea; gkssrudalls@hanyang.ac.kr (K.H.); nicefoxj@hanyang.ac.kr (K.J.); 2 School of Electrical Engineering, Hanyang University, 55 Hanyangdaehak-ro, Sangnok-gu, Ansan-si 15588, Gyeonggi-do, Korea; wer600@hanyang.ac.kr

**Keywords:** point cloud resampling, electric repulsion force, local surface projection

## Abstract

3D point cloud resampling based on computational geometry is still a challenging problem. In this paper, we propose a point cloud resampling algorithm inspired by the physical characteristics of the repulsion forces between point electrons. The points in the point cloud are considered as electrons that reside on a virtual metallic surface. We iteratively update the positions of the points by simulating the electromagnetic forces between them. Intuitively, the input point cloud becomes evenly distributed by the repulsive forces. We further adopt an acceleration and damping terms in our simulation. This system can be viewed as a momentum method in mathematical optimization and thus increases the convergence stability and uniformity performance. The net force of the repulsion forces may contain a normal directional force with respect to the local surface, which can make the point diverge from the surface. To prevent this, we introduce a simple restriction method that limits the repulsion forces between the points to an approximated local plane. This approach mimics the natural phenomenon in which positive electrons cannot escape from the metallic surface. However, this is still an approximation because the surfaces are often curved rather than being strict planes. Therefore, we project the points to the nearest local surface after the movement. In addition, we approximate the net repulsion force using the *K*-nearest neighbor to accelerate our algorithm. Furthermore, we propose a new measurement criterion that evaluates the uniformity of the resampled point cloud to compare the proposed algorithm with baselines. In experiments, our algorithm demonstrates superior performance in terms of uniformization, convergence, and run-time.

## 1. Introduction

With the evolution of 3D scanning technology, in the field of scanning and data acquisition, various types of point clouds are routinely collected by 3D scanners. Researchers use point cloud data in various applications, such as 3D CAD models, medical imaging, entertainment media, and 3D mapping. Despite advances in scanning technology, scanned raw point clouds may have inadequacies such as noise, multilayered surfaces, missing holes, and nonuniformity of distribution, depending on the performance of the scanner. Such poorly organized point clouds have negative effects on downstream applications such as surface reconstruction. Therefore, there have been recent attempts to refine point clouds by eliminating noise, producing evenly distributed data points while retaining the original shape and obtaining high-quality normal information.

Over the past few years, the computer graphics and numerical computation community has intensively studied point cloud resampling techniques. The locally optimal projection (LOP) operator, a popular consolidation method, was proposed by Lipman et al. [1]. They formulated the problem to simultaneously optimize terms that maintain the shape of the input point cloud and widen the distance between the cloud points. This method utilizes only the point locations and does not require the normal vectors. Therefore, this algorithm is robust for point clouds with distorted orientations as well as in cases where the orientations are ambiguous, e.g., when two surfaces lie close to each other. However, in LOP, the density of the output point cloud follows that of the input point cloud, due to which the output point cloud becomes nonuniform.

Huang et al. [2] proposed the weighted LOP (WLOP) operator for initializing normal vector estimation. The WLOP operator improves the LOP by introducing density weights. WLOP compensates sparse areas in a point cloud with density weights. However, this algorithm requires a full pairwise distance calculation as in LOP. Thus, the execution of the algorithm is costly, and moreover, it still does not produce evenly distributed outputs. Additionally, an edge-aware point cloud resampling method was proposed in [3]. This method first resamples the farthest points from the edge by using the LOP operator and gradually resamples the other points near the previously resampled points. Unfortunately, it cannot uniformize the point cloud data effectively as it is based on the LOP algorithm. Liao et al. [4] proposed a feature-preserving LOP (FLOP). They preserved spatial and geometric features by bilaterally weighting them, and the speed of the algorithm was improved by using kernel density estimates. However, it is based on the LOP and still suffers from the limitation that the density of the resulting point cloud follows that of the input point cloud. Preiner et al. [5] adopted a continuous expression of the LOP and WLOP operators and achieved a remarkable reduction of the run time by using a Gaussian mixture to describe the input point cloud density. However, this algorithm is developed as a point cloud meshing method and cannot be used for point cloud resampling. In addition, the centroidal Voronoi tessellation (CVT), which was originally proposed for remeshing polygon meshes [6,7,8,9], was utilized for point cloud resampling by Chen et al. [10]. However, this requires an explicit calculation of the restricted Voronoi cell (RVC) [11], which is computationally more involved.

In view of these advances, we propose a resampling algorithm that is focused on evenly distributing the point cloud. The first key contribution of this paper is the proposal of a point cloud uniformization method based on a simple simulation of electrons on a virtual metallic surface. Here, we consider the electric and damping forces in the simulation. The damping formulation is similar to introducing momentum in mathematical optimization [12], which can facilitate stable convergence. In this process, we compute virtual local surfaces and restrict the repulsion forces to them to prevent movements in the normal directions. When calculating the repulsion forces, we use the kd-tree-based *K*-nearest neighborhood for each point, which is introduced for the speedy execution of our algorithm. The second contribution is proposing a novel measure for quantifying the uniformity of a point cloud. The intuition behind the measure is to evaluate the variance in the local density of a point cloud.

The advantages of our algorithm are that it is simple and intuitive to implement and exhibits outstanding uniformization performance. Furthermore, it exhibits fast and stable convergence thanks to the damping term. From our experiments, one can confirm that our algorithm demonstrates superior uniformity performance compared to the LOP and WLOP algorithms. Furthermore, we provide experiments for various parameter settings, which show that the proposed method is not very sensitive to the change of parameters.

The rest of the paper is organized as follows. Section 2 presents the proposed resampling algorithm that can resample a uniformly distribute point cloud from an unevenly distributed input. In Section 3, we report the experimental results of the proposed method. The uniformity measure for quantifying the quality of the resampled point clouds is also presented here. Section 4 provides the conclusion of the paper.

## 2. Proposed Method

### 2.1. Notations and System Overview of Point Cloud Resampling

The goal of this paper is to resample the input point cloud uniformly while retaining the shape of the given point cloud. Before presenting the details of our algorithm, we define the notations used in this paper. The input point cloud is represented by $P=\left[P_{1},\ldots ,P_{i},\ldots ,P_{N_{P}}\right]\in R^{N_{P}\times 3}$. The input point cloud *P* is a matrix that has $N_{P}$ points, and each point of the point cloud is represented by $P_{i}\in R^{1\times 3},i\in 1,\ldots ,N_{P}$. Even though the point cloud does not have any order, we consider *P* as a matrix by stacking the points. In addition, *P* is assumed to be that its centroid is at the origin, and it is appropriately scaled to ease parameter tuning. $Q^{t}=\left[Q_{1}^{t},\ldots ,Q_{j}^{t},\ldots ,Q_{N_{Q}}^{t}\right]\in R^{N_{Q}\times 3}$ represents the iteratively resampled result of the input point cloud *P*. It is a matrix with $N_{Q}$ points. $V_{j}^{t}\in R^{1\times 3},j\in 1,\ldots ,N_{Q}$ represents the velocity of the iteratively moving point $Q_{j}^{t}$. The velocities determine the amount of movement from $Q_{j}^{t}$ to $Q_{j}^{t+1}$, which is described in detail in Section 2.3. $N_{A}^{B}$ denotes the normal vector ($R^{1\times 3}$) of the point cloud B at query point A.

ϕ(·,·,·) represents a function that obtains the *K*-nearest neighbor points. The first argument represents a query point, the second argument represents a reference point cloud matrix, and the final argument represents *K* of the *K*-nearest neighbor points. For example, $\phi (Q_{j}^{t},P,K)$ is the *K*-nearest neighbor points of query point $Q_{j}^{t}$ in the reference point cloud *P*. Similar to the above terms, we represent these points as a matrix that has $R^{K\times 3}$ dimensions by stacking the points. In addition, to define the *k*th point of the neighbor points, we define $\phi_{k}\left(\right)$ as the *k*th point of the output neighbor points ϕ(). For example, $\phi_{k}\left(Q_{j}^{t},P,K\right)$ denotes the *k*th neighbor point of the query point $Q_{j}^{t}$, which is obtained from the reference point cloud matrix *P*. This results in a vector with dimensions $R^{1\times 3}$. We use these extracted neighbor points to compute the electric force as well as the local tangent plane of the input point cloud. In addition, a projection function ψ(·,·) is also defined, which is used to suppress surface approximation errors. We discuss this in detail in Section 2.2. In addition, for our physical simulation system, we define an electric force $F_{q}^{t}$ that mimics one between electrons in real world, to move the points iteratively. $F_{q}$ denotes the net repulsion force of the query point $Q_{q}$, which is an $R^{1\times 3}$-dimensional vector. The detailed description of $F_{q}$ is discussed in detail in Section 2.2.

**Figure 1:**
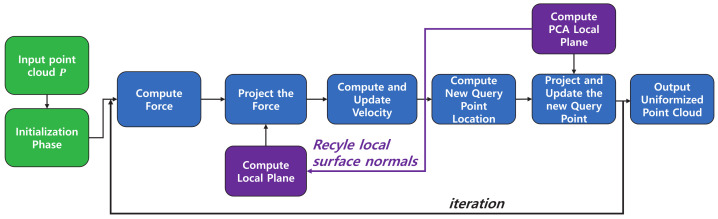
Overview of point cloud resampling algorithm. The input point cloud *P* is assumed to be zero-centered and rescaled. First, the resampled point cloud $Q^{0}$, velocity $V^{0}$, and the normal vectors $N_{Q^{0}}^{P}$ of the local tangent plane are initialized. In each iteration, we perform the following procedures: We compute the *K*-nearest neighbors from $Q^{t-1}$ to calculate the net electric force. Then, the normal vectors of the local tangent planes, calculated in the previous iteration, are used to project the forces to the local surfaces. The next velocities and the new query point cloud $Q^{t}$ are computed based on the forces additionally modified with damping terms. Then, we obtain the *K*-nearest neighbor for the updated point cloud $Q^{t}$ and calculate the local tangent planes. To prevent $Q^{t}$ from diverging, we project it using these new tangent planes. These planes can be reused in the next iteration to project electric forces for efficiency. After the iteration converges, the final output point cloud is rescaled to the original scale and is relocated to have the original center point.

The overview of the proposed method is shown in Figure 1. The input point cloud is first preprocessed to be zero-centered and have a proper scale. Subsequently, we initialize the resampled point cloud $Q^{0}$ to the preprocessed input point cloud *P* and the velocity of each point $V_{q}^{0}$ to zero. Then, the local tangent surface normal vector $N_{Q_{q}^{0}}^{P}$ is initialized by the principal component analysis (PCA) [13] of the *K*-nearest neighbor of $Q^{0}$.

In each iteration, the neighbor points of each query point $Q_{q}^{t-1}$ are used to calculate the net electric repulsion forces. To mimic the physical characteristics of an electron moving on a metallic surface, we need to restrict the net electric force upon $Q_{q}^{t-1}$ to lie on the local tangent plane. This is achieved by projecting the net force based on the local surface normal $N_{Q_{q}^{t-1}}^{P}$. The projected electric repulsion force has only a tangential component on the local plane of each query point. The induced electric repulsion force between the neighbor points and query point causes the query point to move away from its neighbors. Using the induced electric force and a damping term based on the previous velocity $V_{q}^{t-1}$, the new acceleration $a_{q}^{t}$ and velocity $V_{q}^{t}$ are derived. Using $V_{q}^{t}$, we move the query points $Q_{q}^{t-1}$ to $Q_{q}^{t}$.

**Figure 2:**
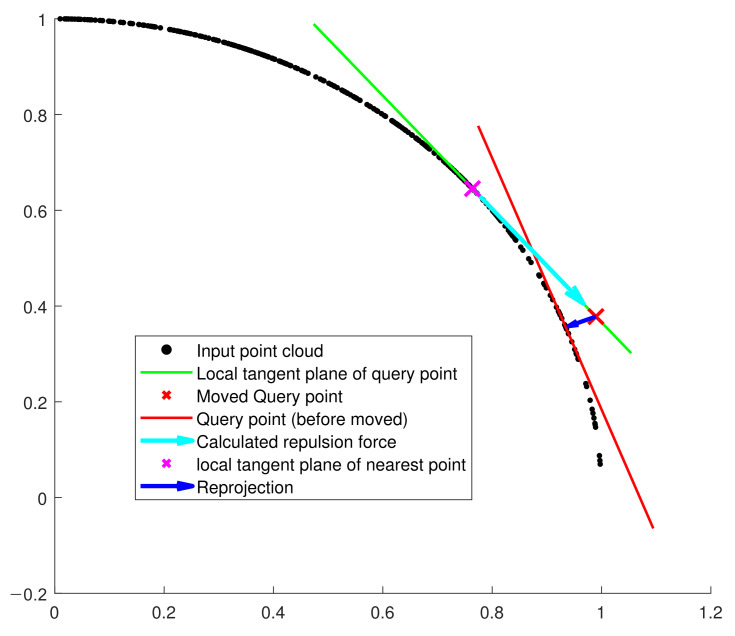
PCA projection restrains the surface approximation error when moved points shift away from the input point cloud’s surface. By using the PCA projection, we project the moved points to the nearest local plane.

However, approximating the virtual local surface as a plane rather than a curved surface makes the moved points $Q_{q}^{t}$ shift away from the nearest local surface. This approximation error is demonstrated in Figure 2. As we can see here, it is simply solved by projecting $Q_{q}^{t}$ to the nearest surface. For this projection, we use the *K*-nearest neighbors of $Q_{q}^{t}$ in the input point cloud *P* to calculate the normal vector $N_{Q_{q}^{t}}^{P}$. To reduce the computational burden, this normal vector is recycled in the next iteration to project the repulsion force.

This whole process is repeated iteratively until convergence. After completing the above iterations, the output point cloud is rescaled to the original size and is relocated to have the original center points. The details of each step are explained in the following sections.

### 2.2. Suppressing Normal Components in Repulsion Forces

In this section, we discuss the repulsion force of electron points lying on the surface of the input point cloud. As mentioned above, we mimic the fact that when electrons are placed on a metallic surface, the electrons cannot escape from the metallic surface. They move based on the repulsion between each other and eventually spread evenly. To simulate this situation, we need to restrict the repulsion forces of the query points to possess only the tangential component along the local plane.

**Figure 3:**
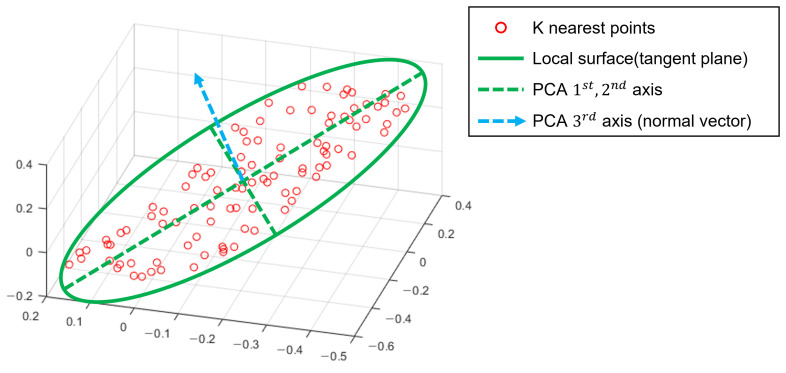
Conceptual image of PCA-based local surface extraction. In a 3D space, the normal vector of the plane is the 3rd eigenvector of the PCA result.

To achieve the above requirement in this paper, any given repulsion force is projected to the local tangent plane based on the projection function ψ(·,·). The first argument of the projection function ψ(·,·) represents the force vector of the query point, and the second argument denotes the normal vector that represents the corresponding local tangent plane. The normal vector is computed using the PCA of the *K*-nearest neighbors of the query point in the input point cloud *P*. We signify the normal vector of point cloud B at query point A as $N_{A}^{B}$. Figure 3 shows an example of the PCA surface approximation.

To calculate the tangent plane (i.e., the corresponding normal vector, in fact) given a query point $Q_{q}^{t-1}$, we first compute the deviations of the *K*-nearest neighbor points from the center of the neighbor points as follows:

(1) $$\Delta_{q,k}^{t-1}=\phi_{k}\left(Q_{q}^{t-1},P,K\right)-\frac{1}{K}\sum_{k=1}^{K}\phi_{k}\left(Q_{q}^{t-1},P,K\right).$$

Then, we compute the covariance matrix *C* using Equation (2).

(2) $$C_{Q_{q}^{t-1}}^{P}=\sum_{k=1}^{K}{\Delta_{q,k}^{t-1}}^{⊤}\Delta_{q,k}^{t-1}.$$

The computed covariance matrix is decomposed using singular value decomposition by Equation (3), and we obtain the normal vector $N_{Q_{q}^{t-1}}^{P}$ of the local tangent plane of the query point $Q_{q}^{t-1}$, i.e., the transposed version of the third column of $W_{Q_{q}^{t-1}}^{P}$.

(3) $$C_{Q_{q}^{t-1}}^{P}=U_{Q_{q}^{t-1}}^{P}D_{Q_{q}^{t-1}}^{P}{W_{Q_{q}^{t-1}}^{P}}^{⊤}.$$

Finally, Equation (4) projects a given net repulsion force $F_{q}^{t}$ of query point based on the normal vector $N_{Q_{q}^{t-1}}^{P}$:

(4) $$\psi \left(F_{q}^{t},N_{Q_{q}^{t-1}}^{P}\right)=F_{q}^{t}-F_{q}^{t}{N_{Q_{q}^{t-1}}^{P}}^{⊤}N_{Q_{q}^{t-1}}^{P}.$$

### 2.3. Moving Points Using Physical System of Electric Forces

In this section, we discuss the simulation system for manipulating electrons. The net electric force of the query point $Q_{q}^{t-1}$ is defined by Equation (5). Here, $k_{e}$ is the electric force constant.

(5) $$F_{q}^{t}=k_{e}\sum_{k=1}^{K}\frac{Q_{q}^{t-1}-\phi_{k}\left(Q_{q}^{t-1},Q^{t-1},K\right)}{|Q_{q}^{t-1}-\phi_{k}\left(Q_{q}^{t-1},Q^{t-1},K\right){|}^{3}}.$$

As explained in the previous section, we project the repulsion force to the local tangent plane to restrain the electric point on the virtual metallic surface using Equation (6).

(6) $$F_{q}^{{}^{\prime }t}=\psi \left(F_{q}^{t},N_{Q_{q}^{t-1}}^{P}\right).$$

In addition, the electron not only moves due to the electric repulsion forces of the neighbor points but is also affected by the damping force. Therefore, the new repulsion force with damping on $Q_{q}^{t-1}$ is defined as $F_{q}^{{}^{\prime }t}-\delta V^{t-1}$. δ denotes the damping ratio. The acceleration of the query point $a_{q}^{t}$ is defined by Equation (7).

(7) $$m_{q}a_{q}^{t}=F_{q}^{{}^{\prime }t}-\delta V^{t-1}.$$

The updated velocity of $Q_{q}^{t}$ is calculated using Equation (8). It is simply computed by combining the previous velocity of the query point $Q_{q}^{t}$ and the amount of change in velocity due to the total force during the time interval Δt.

(8) $$V_{q}^{t}=V_{q}^{t-1}+a_{q}^{t}\Delta t.$$

Using this velocity, the new position of the electron is calculated as

(9) $$Q_{q}^{t}=Q_{q}^{t-1}+V^{t}\Delta t.$$

The above equations can be simplified to obtain concise update equations. By combining (7) and (8), we obtain

(10) $$V_{q}^{t}=\left(1-\frac{\delta }{m_{q}}\Delta t\right)V_{q}^{t-1}+\frac{\Delta t}{m_{q}}F_{q}^{{}^{\prime }t}.$$

Here, if we define a new variable as $V_{q}^{{}^{\prime }t}≜V_{q}^{t}\Delta t$ and assume that the initial velocity $V_{q}^{0}$ is zero, Equation (10) becomes

(11) $$\begin{array}{cc} V_{q}^{{}^{\prime }t} & =\left(1-\frac{\delta }{m_{q}}\Delta t\right)V_{q}^{{}^{\prime }t-1}+\frac{\Delta t^{2}}{m_{q}}F_{q}^{{}^{\prime }t}\\ \; & =\left(1-\frac{\delta }{m_{q}}\Delta t\right)V_{q}^{{}^{\prime }t-1}+k_{e}\frac{\Delta t^{2}}{m_{q}}\psi \left(\sum_{k=1}^{K}\frac{Q_{q}^{t-1}-\phi_{k}\left(Q_{q}^{t-1},Q^{t-1},K\right)}{|Q_{q}^{t-1}-\phi_{k}\left(Q_{q}^{t-1},Q^{t-1},K\right){|}^{3}},N_{Q_{q}^{t-1}}^{P}\right)\\ \; & ≜\alpha V_{q}^{{}^{\prime }t-1}+\beta \psi \left(\sum_{k=1}^{K}\frac{Q_{q}^{t-1}-\phi_{k}\left(Q_{q}^{t-1},Q^{t-1},K\right)}{|Q_{q}^{t-1}-\phi_{k}\left(Q_{q}^{t-1},Q^{t-1},K\right){|}^{3}},N_{Q_{q}^{t-1}}^{P}\right). \end{array}$$

Note that all parameters are abbreviated into α and β. Similarly, Equation (9) becomes:

(12) $$Q_{q}^{t}=Q_{q}^{t-1}+V_{q}^{{}^{\prime }t}.$$

Equations (11) and (12) are the final forms of the proposed update equations. Note that this corresponds to the momentum update form in mathematical optimization.

We set the parameters α and β to 0.9 and $10^{-8}$, respectively. The parameter α is strongly related to the damping ratio δ, which indicates the extent to which the previous velocity $V_{q}^{t-1}$ is discounted. β is related to the electric force constant $k_{e}$. The reason behind the small β is that, due to the normalization preprocessing, the distances between points become very small and thus $1/r^{2}$ becomes relatively high.

### 2.4. PCA Projection to Restrain Surface Approximation Error

As using local tangent planes for projecting the electric forces is an approximation of local surface which is possibly curved, the points moved by this projected forces can shift away from the surface. Therefore, it is necessary to project the relocated electron as well to the nearest local plane. We approximate the nearest local tangent plane at the new location with the *K* nearest points of the input point cloud. We demonstrate this concept in Figure 2.

The PCA projection for restraining the surface approximation error is similar to the process that projects repulsion forces to each local plane, as described in Section 2.2. The difference here is that the center of the local surface is also required in addition to the normal direction, because we have to calculate the projected *position* of an electron unlike the previous case where the projected directional component of the force is calculated. Accordingly, we define another projection function ω(·,·,·) for this purpose. Similar to ψ(), the first and the second arguments are the query point and the normal vector of the local surface, respectively. The third argument is the center of the local surface, and we use the mean of the *K*-nearest neighbor points for this argument.

By using Equations (13) and (14), we obtain the *K*-nearest neighbors of the moved point $Q_{q}^{t}$ in the input point cloud *P* and calculate the corresponding covariance matrix.

(13) $$\Xi_{q,k}^{t-1}=\phi_{k}\left(Q_{q}^{t},P,K\right)-\frac{1}{K}\sum_{k=1}^{K}\phi_{k}\left(Q_{q}^{t},P,K\right).$$

(14) $$C_{Q_{q}^{t}}^{P}=\sum_{k=1}^{K}{\Xi_{q,k}^{t-1}}^{⊤}\Xi_{q,k}^{t-1}.$$

Using SVD, the surface normal $N_{Q_{q}^{t}}^{P}$ is extracted. $N_{Q_{q}^{t}}^{P}$ is the transpose of the third column of $W_{Q_{q}^{t}}^{P}$.

(15) $$C_{Q_{q}^{t}}^{P}=U_{Q_{q}^{t}}^{P}D_{Q_{q}^{t}}^{P}{W_{Q_{q}^{t}}^{P}}^{⊤}.$$

Moreover, the center of the local plane is calculated as

(16) $$b_{Q_{q}^{t}}^{P}=\frac{1}{K}\sum_{k=1}^{K}\phi_{k}\left(Q_{q}^{t},P,K\right).$$

Finally, we project the query point on the approximated plane represented by $N_{Q_{q}^{t}}^{P}$ and $b_{Q_{q}^{t}}^{P}$. The resampled point $Q_{q}^{t}$ is updated with the projected point.

(17) $$Q_{q}^{t}\leftarrow \omega \left(Q_{q}^{t},N_{Q_{q^{t}}}^{P},b_{Q_{q}^{t}}^{P}\right)=Q_{q}^{t}-\left(Q_{q}^{t}-b_{Q_{q}^{t}}^{P}\right){N_{Q_{q}^{t}}^{P}}^{⊤}N_{Q_{q^{t}}}^{P}.$$

The detailed summary of the proposed method is presented in Algorithm 1.
**Algorithm 1** Proposed resampling algorithm1:Preprocess the input point cloud *P*, so that it is zero-centered and has a proper scale.2:Initialize resampled point cloud $Q^{0}$ using *P*.3:Initialize $V^{{}^{\prime }0}$ as zero and $N_{Q^{0}}^{P}$ based on the local PCA surface approximation of initial point cloud $Q^{0}$ by Equations (1)–(3)4:Initialize *t* to one.5:Find the neighbor points of $Q_{q}^{t-1}$ in $Q^{t-1}$ and net repulsion forces $F_{q}^{t}$ on $Q_{q}^{t-1}$ by using the neighbor points by Equation (5)6:Project the repulsion forces on the local surface by Equation (6)7:Using the projected repulsion forces and $V^{{}^{\prime }t-1}$, the new values of $Q^{t}$ and $V^{{}^{\prime }t}$ are computed using Equations (11) and (12).8:Project $Q^{t}$ to the input point cloud *P* for restraining surface approximation error by Equation (17).9:Increase *t* by one.10:Repeat steps 5–9 until the maximum iteration is reached.11:Rescale the last resampled result to the original scale and relocate the rescaled point cloud to have the original center position.

## 3. Experimental Results

### 3.1. Parameter Settings

Here, we explain the parameter settings for the proposed method. As mentioned earlier, α and β were set to 0.9 and $10^{-8}$, respectively. The number of neighbor points *K* used for approximating the local tangent plane was set to 15. All the input point clouds were preprocessed as follows: their centroids were translated to the origin, and they were rescaled (uniformly in all directions) so that they had unit length on the *x* axis. The original scale and translation were restored at the final stage of the proposed method.

In this paper, we used LOP [1] and WLOP [2] as the compared methods. The parameters of each algorithm were fixed to the ones proposed by the corresponding authors. To make a fair comparison, we fixed the parameters of our method for all the experiments.

All algorithms were executed for 50 iterations for fair comparison. All experiments were conducted on an Intel(R) Xeon(R) CPU E5-2687W v3 @ 3.10 GHz.

### 3.2. Data Sets

We used five well-known point cloud data from Visionair [14]. To generate unevenly distributed point cloud data, we perturbed these point clouds by adding white Gaussian noise to all coordinates. (We call this omnidirectional noise, hereafter.) The power of the white Gaussian noise was set to −55 dBW. The corrupted point clouds were used as inputs to the compared algorithms. We also conducted a tangential noise experiment by adding noise without any normal directional components. The resulting noisy point clouds retained the shape of the original point cloud but differed only in terms of surface uniformity. The tangential noise was created by first generating points with omnidirectional noise and then projecting them to the local tangential plane. In addition, we also generated cases where there were holes on the surface of the point cloud in order to test the algorithm’s ability under extreme conditions. To generate holes, we selected 30 random points in the input point cloud and removed all the points within a ball with radius 0.05. Additionally, we tested our algorithm for real data. There are many point cloud data sets with real-world 3D scans, such as [15,16,17]. Here, we used the Washington RGB-D Scenes data set [15]. Among the samples in the Washington data set, we used Lemon and Flashlight for our demonstration. These samples have many nonuniform regions as well as aliasing effects due to the limitations of sensors or 3D scanning errors. Moreover, these samples contain only a part of the scanned object because they were captured from one viewpoint.

### 3.3. Proposed Uniformity Measure

To discuss surface uniformity, we must define a measure. We propose a new surface uniformity measure in this paper. The measure is defined as the variation of the number of neighboring points in the point cloud. Here, the neighbor points of a given point are determined as the points within a certain radius. We also normalize the measure by the total number of points in the point cloud. The detailed expression for the measure is given as follows: let ρ(·,·,·) be the neighbor count function. Given a query point, a reference point cloud, and a radius, which are the first, second, and third arguments of ρ, respectively, this function returns the number of neighbor points of the query point within the radius in the reference point cloud. Then, given a point cloud *Q*, the proposed uniformity measure *u* is calculated as

(18) $$u=\frac{1}{\left|Q\right|}\sqrt{\left(E\left[{\left(\rho \left(Q_{q},Q,r\right)-\frac{1}{\left|Q\right|}\sum_{q=1}^{\left|Q\right|}\rho (Q_{q},Q,r)\right)}^{2}\right]\right)}.$$

### 3.4. Point Cloud Resampling Results

**Figure 4:**
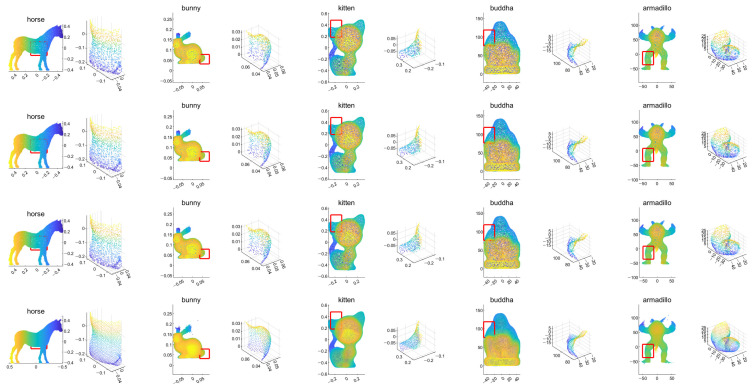
Example results for the tangential noise cases. The first row is the input point cloud, the second row is the resampling result of the LOP algorithm, the third row is that of the WLOP, and the final row is that of the proposed algorithm. The odd columns are the resampled point cloud (from left to right, Horse, Bunny, Kitten, Buddha, and Armadillo), and the even columns are the corresponding enlarged views.

First, we conducted experiments for resampling cases where the numbers of points in the input and output are the same. Figure 4 shows example results for data with tangential noise. Here, we can confirm that the proposed algorithm generally have better uniformization performance than the other algorithms.

**Figure 5:**
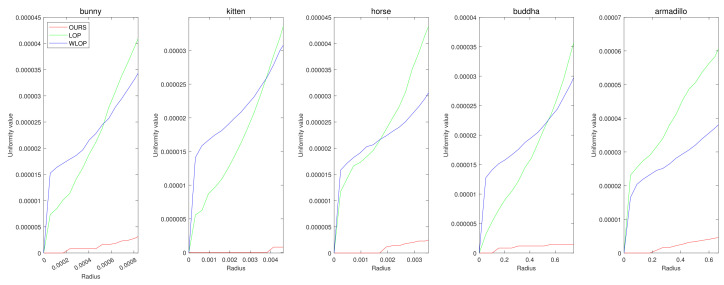
Quantitative results for the tangential noise cases. Each column shows the results of algorithms applied to Horse, Bunny, Kitten, Buddha, and Armadillo. The *x*-axes in the plots indicate the radius of evaluating *u*. The ranges of the radius were determined proportional to the square roots of the ratios between the surface areas of point clouds and the numbers of points.

**Figure 6:**
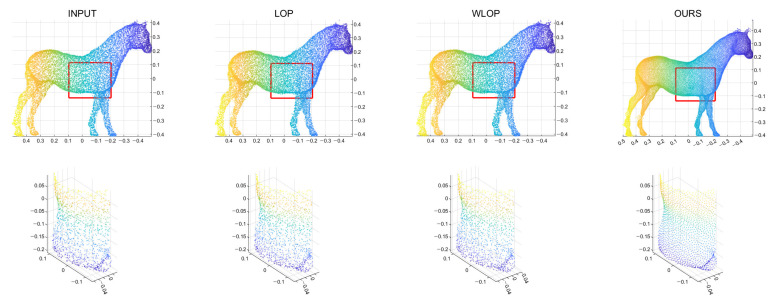
Qualitative results for a tangential noise case (Horse). The second row shows the enlarged views of the red boxes in the first row. The first column shows the input point cloud. The second column shows the result of the LOP. The third column shows that of the WLOP. The last column shows that of the proposed algorithm.

Figure 5 and Figure 6 show the quantitative and qualitative comparisons for the tangential noise case. Here, the maximum ranges of radius (the *x*-axis) of plots in Figure 5 were determined as $2\sqrt{\frac{S}{\pi |Q|}}$, where *Q* is the resampled point cloud and *S* is the corresponding surface area. Since it is difficult to find the exact value of *S*, it was approximately calculated based on the alphaShape function in MATLAB. Here, the proposed method shows considerably better performance than WLOP and LOP, both quantitatively and qualitatively. In the qualitative comparison, the results of LOP and WLOP are barely improved from the input. This shows the disadvantage of these methods, i.e., the results having strong dependence on the input density.

**Figure 7:**
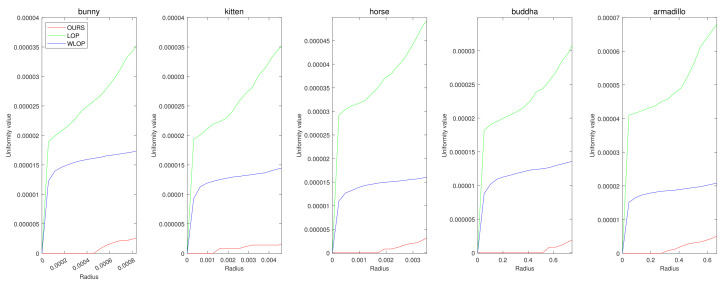
Quantitative results for the omnidirectional noise cases. Each column represents different input data (first column: Horse; second column: Bunny; third column: Kitten; fourth column: Buddha; and fifth column: Armadillo).

**Figure 8:**
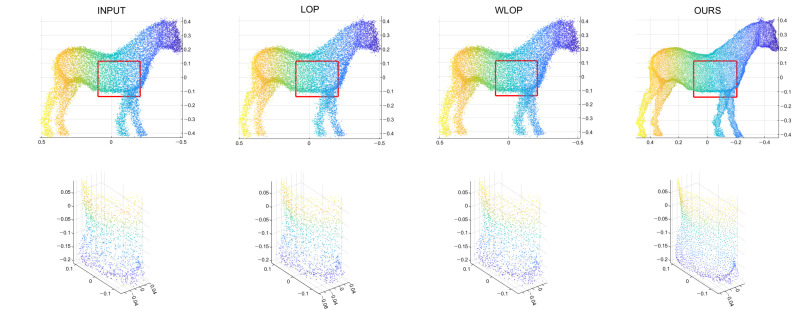
Qualitative results for an omnidirectional noise case (Horse). First column: input point cloud; second column: LOP; third column: WLOP; and fourth column: proposed method. The second row shows enlarged views of the first row.

In the cases with omnidirectional noise, the proposed method again shows outstanding performance as we can see in Figure 7. Figure 8 shows the corresponding qualitative comparison. Here, we can see that the result of the proposed method has significantly smaller normal directional noise than the input and those of the other algorithms.

**Figure 9:**
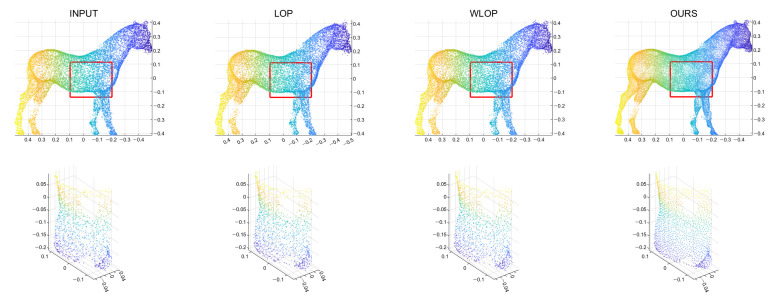
Hole-filling results for the tangential directional noise case (Horse). First column: input point cloud with holes and tangential noise; second column: LOP; third column: WLOP; and fourth column: proposed method. The second row shows enlarged views of the first row.

In addition, we conducted experiments for data with artificially generated missing holes. As mentioned in Section 3.2, we generated missing holes in the point clouds with tangential noise. As we can see in Figure 9, our algorithm exhibits better hole-filling ability than the other algorithms.

**Figure 10:**
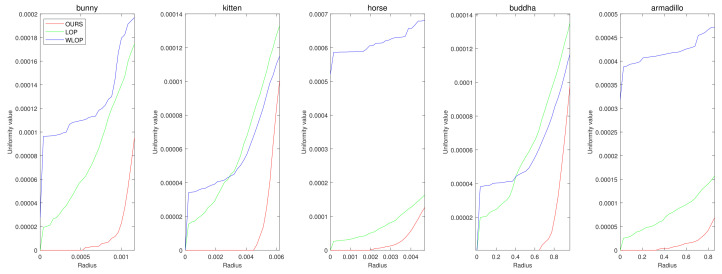
Quantitative results for the tangential noise cases with resampling ratio 0.5. Each column represents different input data (first column: Horse; second column: Bunny; third column: Kitten; fourth column: Buddha; and fifth column: Armadillo).

**Figure 11:**
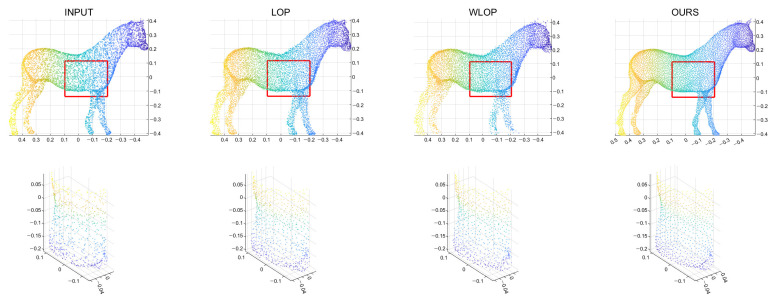
Qualitative results for a tangential noise case with resampling ratio 0.5 (Horse). First column: input point cloud; second column: LOP; third column: WLOP; and fourth column: the proposed method. The second row shows enlarged views of the first row.

**Figure 12:**
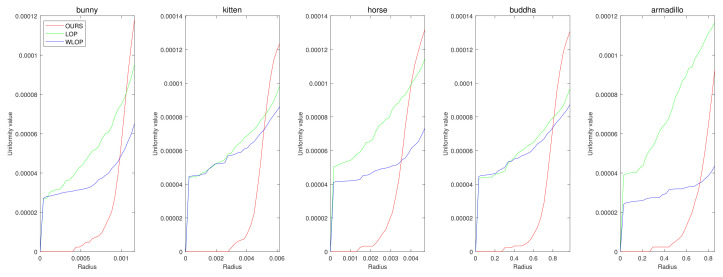
Quantitative results for the omnidirectional noise cases with resampling ratio 0.5. Each column represents different input data (first column: Horse; second column: Bunny; third column: Kitten; fourth column: Buddha; and fifth column: Armadillo).

**Figure 13:**
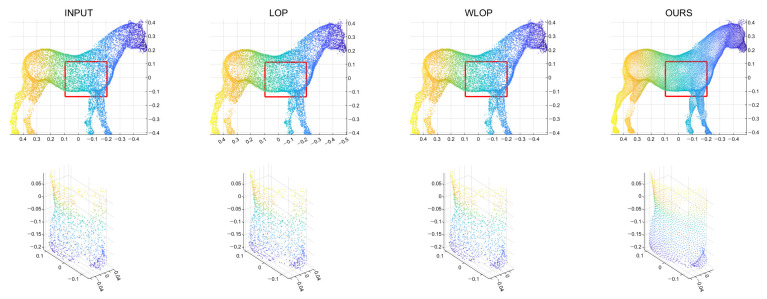
Qualitative results for an omnidirectional noise case with resampling ratio 0.5 (Horse). First column: input point cloud; second column: LOP; third column: WLOP; and fourth column: the proposed method. The second row shows enlarged views of the first row.

We also evaluated the performance of point cloud downsampling and upsampling. For the downsampling experiment, we set the resampling ratio to 0.5. This is achieved by initializing $Q^{0}$ to a randomly subsampled version of the input point cloud. Figure 10 and Figure 11 show the tangential noise case. Similar to the previous experiments, the proposed method shows superior performance to the other algorithms. In the case of omnidirectional noise, there is no apparent winner between the proposed method and WLOP in Figure 12. However, it is clear that the proposed method shows much better performance values for smaller radii. Evaluating *u* with a smaller radius indicates local density better; therefore, the performance for a smaller radius holds much more importance. In this regard, we can say that the proposed method shows much better characteristics. This is apparent in Figure 13, where our method qualitatively outperforms the compared methods.

**Figure 14:**
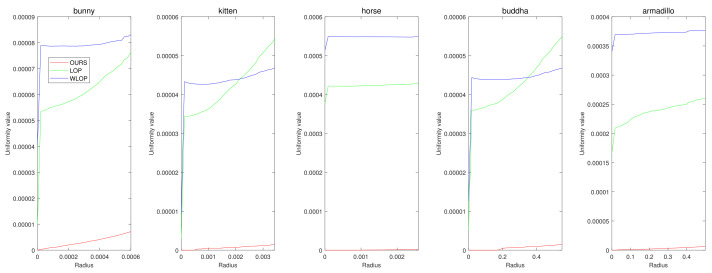
Quantitative results for the tangential noise cases with resampling ratio 2.0. Each column represents different input data (first column: Horse; second column: Bunny; third column: Kitten; fourth column: Buddha; and fifth column: Armadillo).

**Figure 15:**
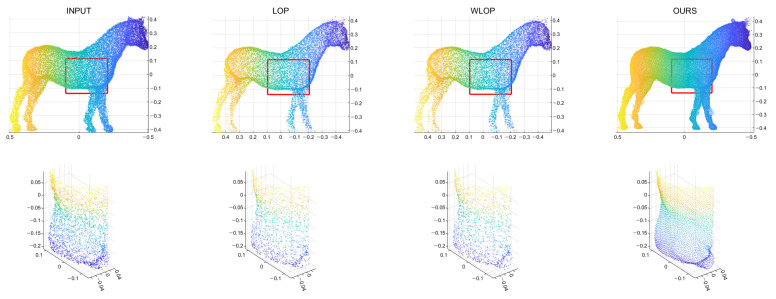
Qualitative results for an tangential noise case with resampling ratio 2.0 (Horse). First column: input point cloud; second column: LOP; third column: WLOP; and fourth column: the proposed method. The second row shows enlarged views of the first row.

For upsampling experiments, we needed to generate an initial $Q^{0}$ that has double the size of the input point cloud. For this, we generated another instance of point cloud by adding Gaussian noise to the input point cloud. Then, we concatenate this to the original input to generate the initial $Q^{0}$. The proposed algorithm stands out in the upsampling case with tangential noise, as can seen in Figure 14. Compared to downsampling, there are wider performance gaps. The qualitative results are shown in Figure 15. The qualitative performance of proposed method is noticeably improved. Moreover, the results of LOP and WLOP seem even more sparse than the input point cloud in this case. This artifact comes from the fact that many of the resampled points are clustered together. These algorithms’ strong dependence on the input density manifests in this phenomenon for upsampling cases.

**Figure 16:**
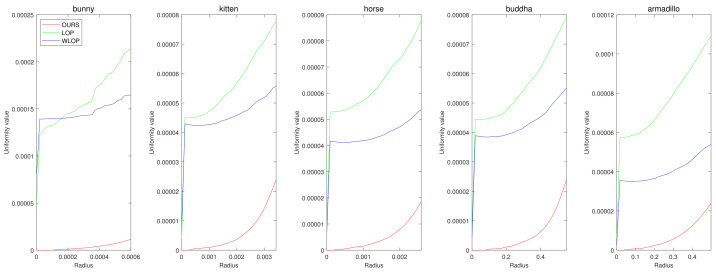
Quantitative results for the omnidirectional noise cases with resampling ratio 2.0. Each column represents different input data (first column: Horse; second column: Bunny; third column: Kitten; fourth column: Buddha; and fifth column: Armadillo).

**Figure 17:**
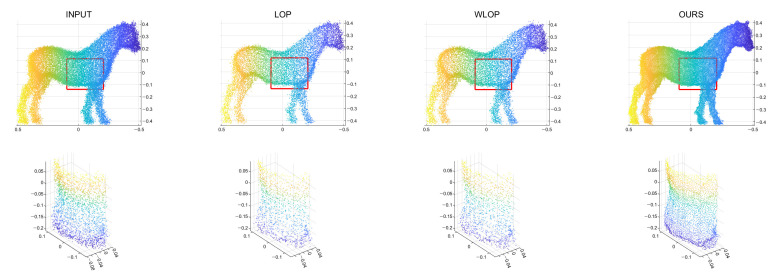
Qualitative results for an omnidirectional noise case with resampling ratio 2.0 (Horse). First column: input point cloud; second column: LOP; third column: WLOP; and fourth column: the proposed method. The second row shows enlarged views of the first row.

The upsampling results with the omnidirectional noise are shown in Figure 16. Again, LOP and WLOP did not work well in this case. These results shows that LOP and WLOP are not appropriate for upsampling. However, the proposed method still shows superb performance. In addition, similar to the resampling cases with omnidirectional noise, the proposed method has better ability to suppress normal directional noise, as shown in Figure 17.

**Figure 18:**
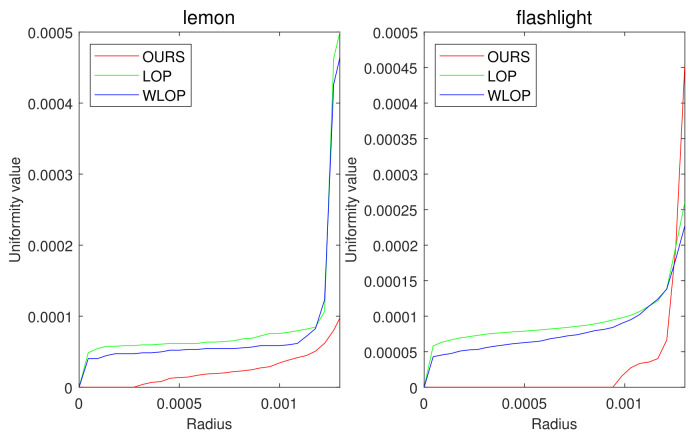
Quantitative result for real data sets. The first and second columns show the uniformity results of each algorithm for Lemon and Flashlight.

**Figure 19:**
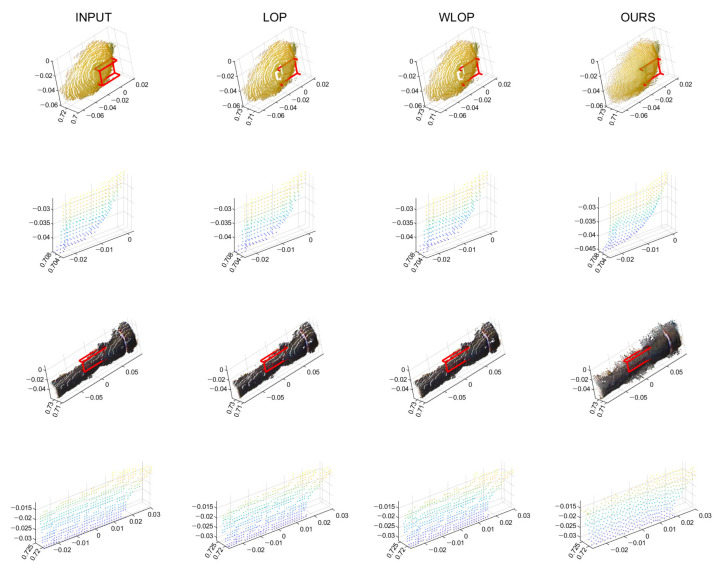
Qualitative results for real data sets. The first row shows the resampled results of Lemon. The second row shows enlarged views of the first row. The third row shows the resampled results of Flashlight. The fourth row shows enlarged views of the third row. First column: input point cloud; second column: LOP; third column: WLOP; and fourth column: proposed method.

As we mentioned above, we have also experimented on real scanned data. In Figure 18, our algorithm performs better than the other algorithms, as expected. In addition, the qualitative results in Figure 19 show that our algorithm can provide a smooth surface to an input with an aliasing problem.

### 3.5. Parameter Tuning

**Figure 20:**
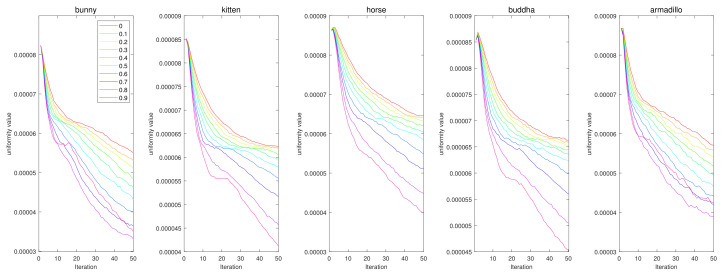
Quantitative performance of the proposed method for various α. The horizontal axis indicates the iteration, and the vertical axis indicates the uniformity value. Each column represents a different input point cloud (first column: Horse, second column: Bunny, third column: Kitten, fourth column: Buddha, and fifth column: Armadillo).

**Figure 21:**
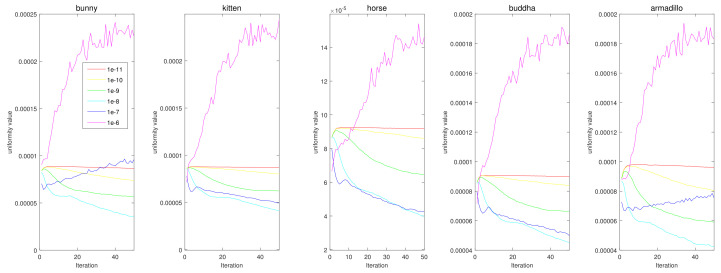
Quantitative performance of the proposed method for various β. The horizontal axis indicates the iteration, and the vertical axis indicates the uniformity value. Each column represents a different input point cloud (first column: Horse, second column: Bunny, third column: Kitten, fourth column: Buddha, and fifth column: Armadillo).

We conducted parameter tuning experiments for α and β. First, in Figure 20, the results show that the case with no momentum (α=0) has the worst results for all data. Interestingly, we can see that the uniformization performance increases as α increases. However, if we set α to one, $V_{q}^{{}^{\prime }t}$ diverges according to Equation (11). Therefore, in this paper, we used α=0.9. In Figure 21, we tested various values for β, and $\beta =10^{-8}$ was the best for most cases.

### 3.6. Running Time and Convergence Results

In this subsection, we tested the running time and convergence of the each algorithm. The run times of 50 iterations for each algorithm are listed in Table 1 for three different resampling ratios with inputs with tangential noise. We tested these algorithms 10 times for all cases and reported the mean of the observed run times. Here, the LOP and the WLOP consume more time because they have quadratic complexity for the pairwise distance calculation. The proposed method is much faster than the other methods most of the time.

**Figure 22:**
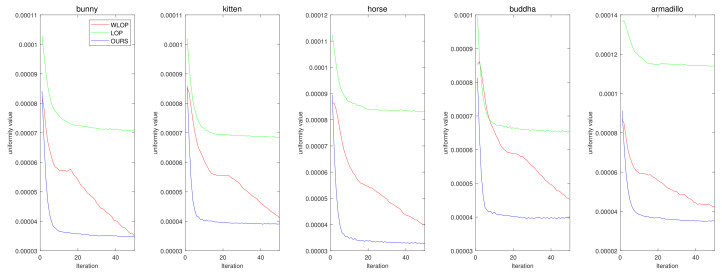
Convergence results of compared methods for the resampling experiment with tangential case. (first column: Horse, second column: Bunny, third column: Kitten, fourth column: Buddha, and fifth column: Armadillo).

In addition, in Figure 22, we tested the convergence of each algorithm. The results shows that our algorithm has superior convergence properties for the Visionair data. This confirms that our algorithm is more stable for resampling input point clouds than the other algorithms.

### 3.7. Discussion on More Complicated Geometries

In this section, we discuss more complicated cases and possible limitations of the proposed method. The proposed method is a numerical method which relies on the local plane assumption. This makes some parameters critical for the success of the algorithm or determines the limitations of the method. Ideally, it is desirable to have small and accurate local planes. Accordingly, there are two dominant factors: the density of the input point cloud and the size of local neighborhoods. The latter is determined by *K* in our algorithm. We might use points within a certain radius instead, but this sometimes can result in having no point at all; therefore, we stick to *K*-nearest neighbors. The above two factors being critical is more or less shared with many other existing numerical resampling methods, including the LOP and WLOP compared in this paper. Even though LOP and WLOP do not directly use *K*-nearest neighbors in their formulations, their update equations still give strong emphasis on the neighboring points.

**Figure 23:**
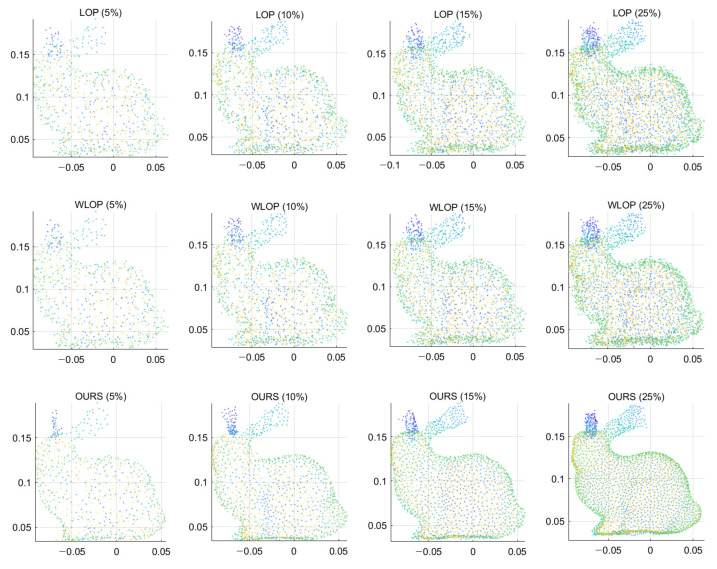
Resampling results of low-density inputs. The input point clouds were generated by randomly subsampling the input data of Figure 5. The percentages in the parentheses represent the amount of subsampling. First row: LOP, second row: WLOP, and third row: proposed method.

If the above assumption, i.e., local neighborhood being accurate and small, is violated, then the proposed method might have some errors. A straightforward example is the input point cloud being too sparse. In this case, we have to sacrifice either the accuracy or the smallness of the local neighborhoods. Sacrificing the former might lose the stability of the local plane estimates, while sacrificing the latter might lose high-frequency details. The proposed method belongs to the latter case (i.e., using *K*-nearest neighbors with a fixed *K*). To demonstrate such a characteristic, we generated sparse input point clouds with extreme subsampling. We applied the resampling methods to these data and set the density of the output identical to the input. In Figure 23, the results show that our algorithm is trying to approximate more areas at fixed *K* as the density of the input point cloud decreases. As a result, the output becomes more smoothed. This trend is less prominent for LOP and WLOP; however, their overall quality is much worse than that of the proposed method.

**Figure 24:**
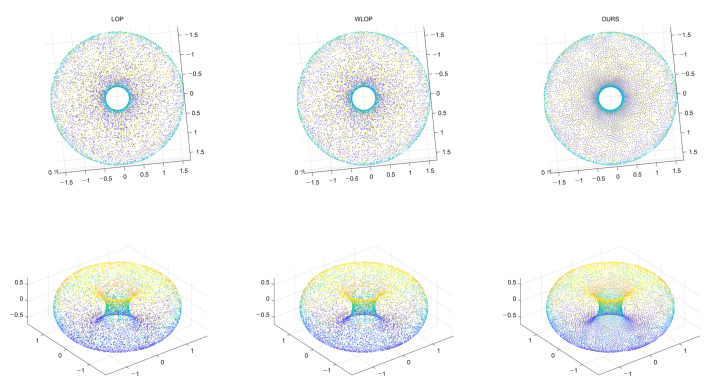
Resampling result of a genus-one shape. Left: LOP, middle: WLOP, and right: proposed method.

Another possible scenario is the shapes of genus one or more. The proposed method can handle shapes of genus one or more; however, this really depends on the size of the local neighborhoods. If the size of a hole is smaller than that of the local neighborhoods, then it is likely that this is considered as a surface with uneven density rather than a hole. Such a case has been already demonstrated in the experiment of Figure 9. Hence, there is a trade-off between the preservation of holes and the stability of resampling. In order to verify that the proposed method can handle a hole properly in the right circumstance, we generated a doughnut-shaped genus one surface. In Figure 24, we can confirm that the hole is well preserved in the resampling result. The obvious reason is that the density of the input point cloud is high enough in this case so that the hole is much larger than the local neighborhoods.

**Figure 25:**
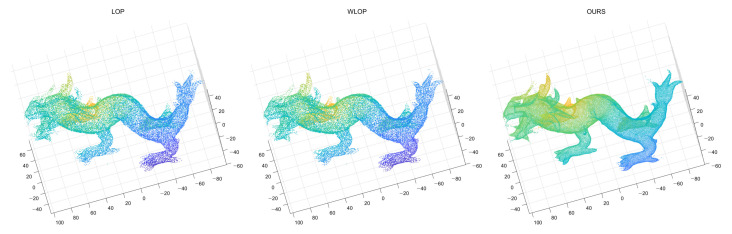
Resampling results of Dragon. (**Left**): LOP, (**Middle**): WLOP, (**Right**): proposed method.

Finally, shapes with sharp regions or high-frequency details can be another source of error for calculating the local neighborhoods. To demonstrate this, we used the Dragon model from the Visionair data set [14]. The results are shown in Figure 25. Here, the proposed method has a few points diverging at the end of sharp regions. For the LOP and WLOP, there are fewer such diverging points, but the errors are more in the form of points becoming scarce around the sharp regions: The density in parts such as the horns of the dragon is much lower than that of the body. Meanwhile, our algorithm has the highest level of uniformity for the given data among the compared methods. Fortunately, the diverging points can be easily fixed through a simple algorithm such as an outlier removal; therefore, we can say that our method is still relevant in these kinds of data.

## 4. Conclusions

We proposed a novel point cloud resampling algorithm based on simulating electrons on a virtual metallic surface. To mimic the movements of electrons on the metallic surface, the proposed method suppresses the normal component of the repulsion forces on the local surface. However, due to the use of a simple plane model for the surface approximation, the points on a possibly curved surface may exhibit some approximation errors. This was resolved by performing point projection to the nearest surface.

## Figures and Tables

**Table 1:** Running times of different algorithms for various input data and resampling ratios. The best results are highlighted in bold.

Resampling Ratio	Method	Horse	Bunny	Kitten	Buddaha	Armadilo
0.5 (Subsampling)	LOP	112.35 s	**57.81** s	96.84 s	108.57 s	112.89 s
	WLOP	156.98 s	144.96 s	153.67 s	141.39 s	118.76 s
	ours	**73.97 s**	75.52 s	**74.73 s**	**55.61 s**	**54.96 s**
1.0 (Resampling)	LOP	435.17 s	424.60 s	437.59 s	406.28 s	296.43 s
	WLOP	585.16 s	559.99 s	584.19 s	549.82 s	428.72 s
	ours	**108.24 s**	**112.36 s**	**111.71 s**	**105.53 s**	**107.21 s**
2.0 (Upsampling)	LOP	752.24 s	763.53 s	748.47 s	705.54 s	743.19 s
	WLOP	1150.53 s	1030.98 s	1083.53 s	1101.86 s	1119.77 s
	ours	**284.78 s**	**219.58 s**	**237.51 s**	**254.56 s**	**280.32 s**

## Data Availability

To obtain the data sets used in this paper, please refer to [14,15].
